# Intralobar pulmonary sequestration displayed as localized emphysema on computed tomography image

**DOI:** 10.1186/s13019-017-0646-9

**Published:** 2017-09-08

**Authors:** Weibo Qi, Junjie Zhao, Guping Shi, Fan Yang

**Affiliations:** Department of Cardio-Thoracic Surgery, The First Hospital of Jiaxing, Jiaxing, 314000 Zhejiang, People’s Republic of China

**Keywords:** Pulmonary, Sequestration, Computed tomography

## Abstract

**Background:**

Pulmonary sequestration is a relatively rare condition in which a systemic artery supplies blood to an abnormal lung tissue and the normal connection with the bronchial tree is absent. It can be displayed as various signs on the computed tomography image, but emphysema is extremely rare.

**Case presentation:**

We describe the case of a 35-year-old man with intralobar pulmonary sequestration that appeared as localized emphysema on the computed tomography image. The 3-D reconstruction revealed the presence of an anomalous feeding artery and the absence of normal connection with the bronchial tree.

**Conclusion:**

We presumed that it was a type between “anomalous systemic arterial supply to the normal lung” and the common type of pulmonary sequestration. Common pulmonary lobectomy was performed and the patient recovered well.

## Background

In pulmonary sequestration, the lung lesion is usually serious, and the pulmonary alveoli have completely lost their normal structure due to the lack of a normal connection with the bronchial tree. Herein, we report a case of pulmonary sequestration in which the lung lesion was relatively slight and was shown as localized emphysema on the computed tomography image.

## Case presentation

The patient, a 35-year-old man who coughed for 1 week was referred to our hospital. He had no history of smoking, and the physical examination and the laboratory investigation showed no particular findings. The computed tomography scan of the chest revealed emphysema localized in the posterior basal segment of the right lower pulmonary lobe. The further three-dimensional computed tomography image showed that the anomalous feeding artery originated from the celiac trunk.

These findings led us to the diagnosis of pulmonary sequestration and because the normal function of respiration was absent in the lesion, we decided to remove the right lower lobe. The thoracoscopic lobectomy of the lung confirmed the existence of an anomalous feeding artery. The patient recovered well and was discharged from the hospital on postoperative day 7. No problems were observed during the 4-year postoperative follow-up period.

## Discussion

Pulmonary sequestration accounts for 0.15–6.40% of all congenital lung malformations [1]. Usually, the condition is difficult to diagnose preoperatively because of the various clinical manifestations presented. Pryce [2] described this disease in 1946. Pulmonary sequestration is divided into two types: intralobar and extralobar. It is noteworthy that Pryce [2] subdivided intralobar pulmonary sequestration into three types. According to his classification, in type 1, there is no abnormal lung tissue. Since no lung sequestration is present in this type, many professionals have recently named it “anomalous systemic arterial supply to the normal lung” to distinguish it from the real pulmonary sequestration. Therefore, pulmonary sequestration is a condition in which a segment or lobe of lung tissue has no bronchial communication with the normal tracheobronchial tree.

**Fig. 1:**
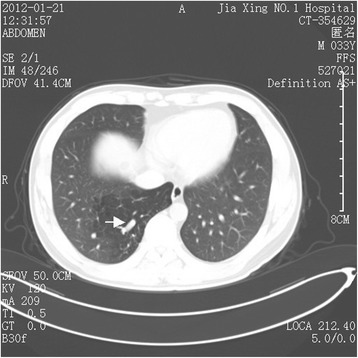
Emphysema localized on the posterior basal segment of the right lobe and the anomalous feeding artery (arrow) located in the abnormal lung tissue

**Fig. 2:**
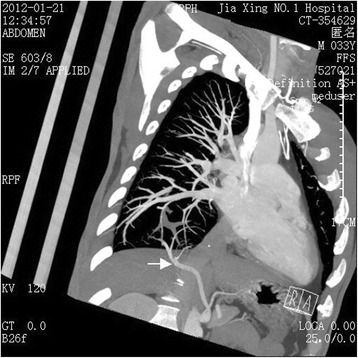
The feeding artery (arrow) enters the right lower lobe of the lung

**Fig. 3:**
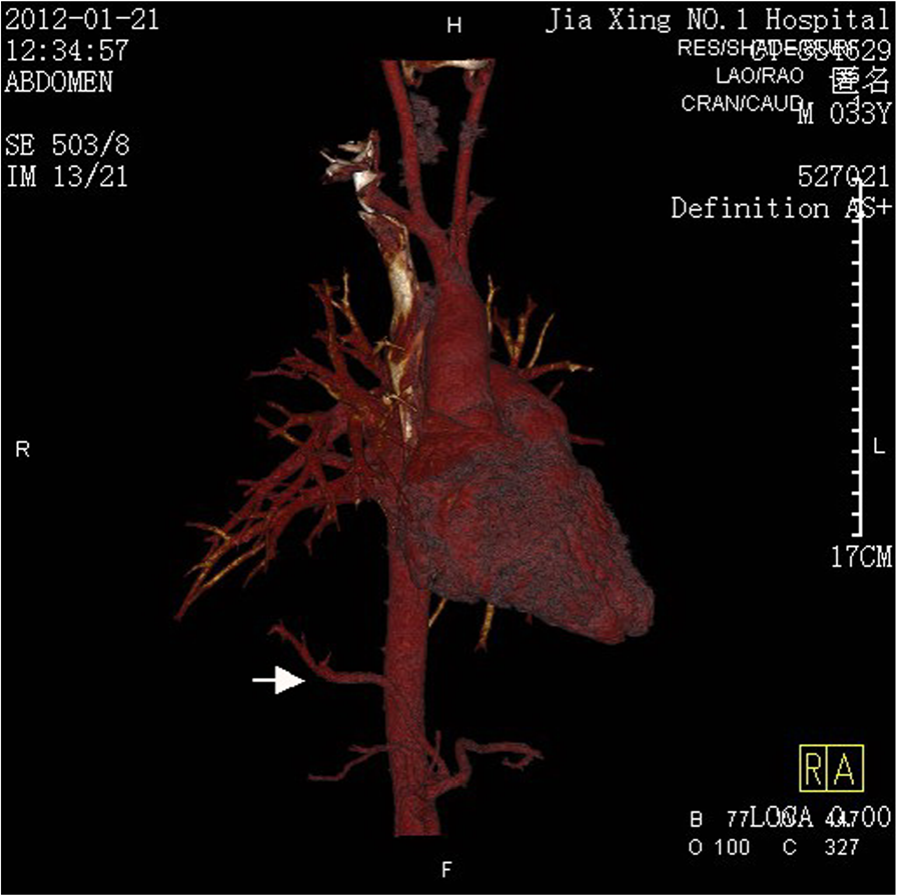
A three-dimensional reconstruction image showing the full view of the feeding artery (arrow) originating from the celiac trunk

**Fig. 4:**
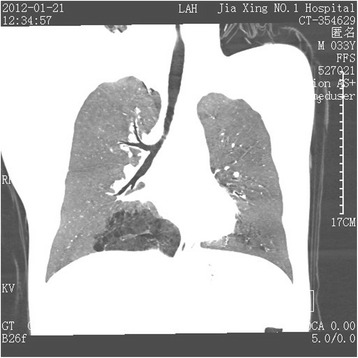
No normal branch of the tracheobronchial tree to the right posterior basal segment was present

Localized emphysema occurs rarely (Fig. 1), and on a computed tomographic image, the abnormal lung tissue could be displayed as any of the following: a cystic lesion, mass, lamellar shadow, bronchiectasis, encapsulated hydrothorax, or atelectasis. In the case described in the present study, the lung lesion was slighter than that in the common type of pulmonary sequestration. Thus, we presumed that it was a type between the previously defined “anomalous systemic arterial supply to the normal lung” and the common type of pulmonary sequestration. Importantly, the finding of the anomalous feeding artery led us to the right diagnosis. It was clearly visible on the computed tomography image that the feeding artery entered the abnormal lung tissue (Fig. 2). The three-dimensional computed tomographic image showed the full view of (Fig. 3). The sagittal view image revealed the absence of the normal branch of the tracheobronchial tree to the posterior basal segment of the right lobe (Fig. 4).

Conventionally, angiography has been the gold standard for the diagnosis of pulmonary sequestration. However, multiplanar CT and 3-dimensional reconstruction have been increasingly replacing this diagnostic method due to their lower invasiveness, better spatial resolution in depicting the vessel anatomy of the sequestrated lung tissue [3, 4], and reduced risk of unexpected bleeding in operation. Although the employment of coil embolization as a promising technique has been reported, some authors, such as Kohei Ando [5], consider it just a solution to deal with the patient of “anomalous systemic arterial supply to the normal lung”.

## Conclusion

At present, pulmonary lobectomy is the most common surgical treatment for pulmonary sequestration. The employment of coil embolization is just a solution to the patient of “anomalous systemic arterial supply to the normal lung”. Furthermore, we also deem that the excision of the abnormal lung lobe is reasonable because it has lost the function of respiration.
